# Keep them flapping: How anglers can reduce stress on flapper skates

**DOI:** 10.1093/conphys/coaf017

**Published:** 2025-03-11

**Authors:** Zhong-Wen Jiang

**Affiliations:** School of Ecology, Shenzhen Campus of Sun Yat-sen University, Shenzhen 518107, People’s Republic of China

Angling-induced stress can trigger severe physiological imbalances in critically endangered flapper skates (*Dipturus intermedius*), particularly at high temperatures and with prolonged fight times. New research led by Scottish scientist Georgina Cole has revealed that metabolic and respiratory acidosis—marked by declining blood pH and bicarbonate, surging lactate and carbon dioxide build-up—can push these animals to their physiological limits.

So, how can anglers help conserve skates?

By analysing the health of 61 skates caught off Scotland’s coast, Cole and colleagues found that anglers can significantly reduce stress on skates by shortening fight times, handling them quickly, and minimizing exposure to warm air. These simple yet crucial practices could mean the difference between survival and post-release mortality.

To assess the physiological toll of angling, Cole and colleagues conducted detailed health checks for these skates, taking blood samples immediately after capture and just before release while monitoring heart and respiratory rates to track key markers of stress (Fig. 1). Prior to release, acoustic tags were implanted into the skates for future tracking.

The study identified metabolic and respiratory acidosis as major stress responses in skates, characterized by drops in blood pH and bicarbonate levels, along with spikes in lactate, PCO_2_ and glucose. The primary culprits? High temperatures, prolonged fight times and exposure to air. Interestingly, smaller skates showed strongest stress responses, suggesting they may be particularly vulnerable. On the bright side, tagging did not appear to have additional negative effects, offering reassurance that scientific tracking programmes can continue without undue harm.

**Figure 1:**
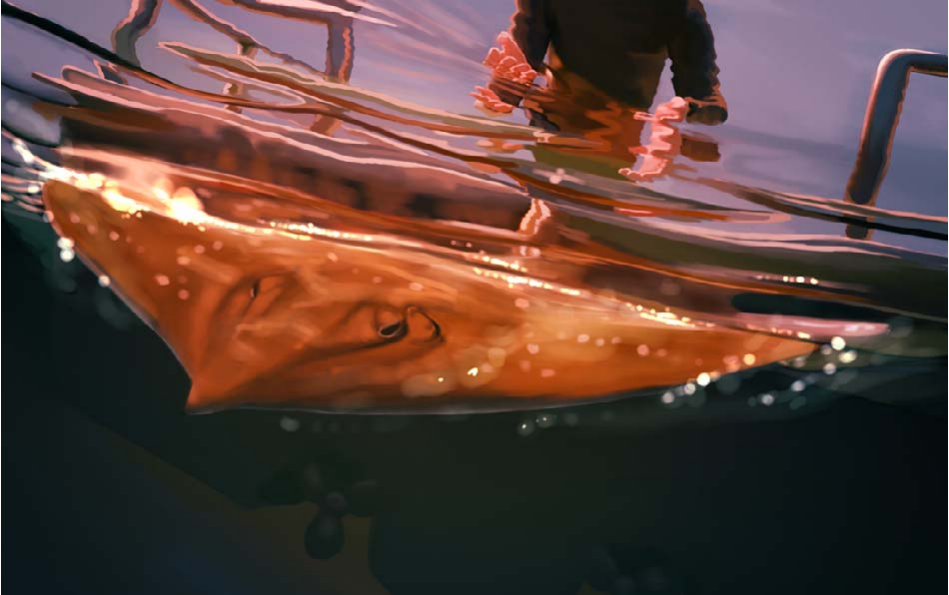
Illustration: Kaitlin Barham (kaitlin.barham@student.uq.edu.au)

However, the impact of angling stress extends beyond individual skates—it may have far-reaching consequences for population health. Sub-lethal effects, such as acidosis and compromised immune function, could make skates more susceptible to predation or long-term fitness declines. This underscores the urgent need for evidence-based handling guidelines to protect these already imperilled animals. Both anglers and conservationists have a key role to play. By refining angling techniques and prioritizing low-stress handling, the community can help ensure that flapper skates—and other vulnerable species—have a fighting chance in an increasingly challenging ocean.

## Notes

**Editor:** Ashleigh Donaldson
